# Subjects With Diabetes Mellitus Are at Increased Risk for Developing Tuberculosis: A Cohort Study in an Inner-City District of Barcelona (Spain)

**DOI:** 10.3389/fpubh.2022.789952

**Published:** 2022-05-23

**Authors:** Violeta Antonio-Arques, Josep Franch-Nadal, Antonio Moreno-Martinez, Jordi Real, Àngels Orcau, Didac Mauricio, Manel Mata-Cases, Josep Julve, Elena Navas Mendez, Rai Puig Treserra, Joan Barrot de la Puente, Joan Pau Millet, Jose Luis Del Val García, Bogdan Vlacho, Joan A. Caylà

**Affiliations:** ^1^DAP-Cat Group, Unitat de Suport a la Recerca Barcelona, Fundació Institut Universitari per a la Recerca a l'Atenció Primària de Salut Jordi Gol i Gurina (IDIAPJGol), Barcelona, Spain; ^2^Primary Health Care Center Bordeta Magòria, Gerència d'Atenció Primària Barcelona Ciutat, Institut Català de la Salut, Barcelona, Spain; ^3^Primary Health Care Center Raval Sud, Gerència d'Atenció Primària Barcelona Ciutat, Institut Català de la Salut, Barcelona, Spain; ^4^CIBER of Diabetes and Associated Metabolic Diseases (CIBERDEM), Instituto de Salud Carlos III (ISCIII), Madrid, Spain; ^5^Department of Infectious Diseases, Hospital Clínic de Barcelona, Barcelona, Spain; ^6^CIBER of Epidemiology and Public Health (CIBERESP), Instituto de Salud Carlos III (ISCIII), Madrid, Spain; ^7^Epidemiology Service, Agència de Salut Pública de Barcelona, Barcelona, Spain; ^8^Department of Endocrinology and Nutrition, Hospital Universitari de la Santa Creu i Sant Pau, Barcelona, Spain; ^9^Department of Medicine, University of Vic—Central University of Catalonia, Barcelona, Spain; ^10^Primary Health Care Center La Mina, Gerència d'Atenció Primària Barcelona Ciutat, Institut Català de la Salut, Barcelona, Spain; ^11^Department of Biochemistry, Institut de Recerca de l'Hospital de la Santa Creu i Sant Pau, Barcelona, Spain; ^12^Fundació Institut Universitari per a la Recerca a l'Atenció Primària de Salut Jordi Gol i Gurina (IDIAPJGol), Barcelona, Spain; ^13^Primary Health Care Center Doctor Jordi Nadal, Gerència d'Atenció Primària Girona Ciutat, Institut Catala de la Salut, Salt, Spain; ^14^Unitat d'Avaluació, Sistemes d'informació i Qualitat, Gerència d'Àmbit d'Atenció Primària Barcelona Ciutat, Institut Català de la Salut, Barcelona, Spain; ^15^Foundation of the Tuberculosis Research Unit of Barcelona, Barcelona, Spain

**Keywords:** diabetes mellitus, tuberculosis, incidence, diabetes complications, *Mycobacterium tuberculosis* infection, alcohol abuse

## Abstract

**Background:**

Tuberculosis is the leading cause of mortality from lung infectious disease worldwide in recent years, and its incidence has re-emerged in large cities in low-incidence countries due to migration and socioeconomic deprivation causes. Diabetes mellitus and tuberculosis are syndemic diseases, with diabetes being considered a risk factor for developing tuberculosis.

**Objective:**

To investigate whether diabetic patients were at increased risk of tuberculosis living in an inner-district of a large city of northeastern Spain.

**Methods:**

Observational matched retrospective cohort study based on clinical records from the population of the lowest socioeconomic status in Barcelona (Ciutat Vella district). A cohort including patients with type 1 and type 2 diabetes mellitus in 2007 and new cases until 2016 (8004 subjects), matched 1:1 by sex and age with a non-diabetic cohort. Follow-up period was until December 31st 2018. We evaluated the risk of developing tuberculosis in diabetic patients compared to non-diabetic patients during the follow up period. We used time-to-event analysis to estimate the incidence of tuberculosis, and competing risks regression by clusters and conditional Cox regression models to calculate the hazard ratio (HR) and its 95% confidence intervals (CI).

**Results:**

Among the 16,008 included subjects, the median follow-up was 8.7 years. The mean age was 57.7 years; 61.2% men and 38.8% women in both groups. The incidence of tuberculosis was 69.9 per 100,000 person-years in diabetic patients, and 40.9 per 100,000 person-years in non-diabetic patients (HR = 1.90; CI: 1.18–3.07). After adjustment for the country of origin, chronic kidney disease, number of medical appointments, BMI, alcoholism and smoking, the risk remained higher in diabetic patients (1.66: CI 0.99–2.77). Additionally, subjects from Hindustan or with a history of alcohol abuse also showed a higher risk of developing tuberculosis (HR = 3.51; CI:1.87–6.57, and HR = 2.73; CI:1.22–6.12 respectively).

**Conclusion:**

People with diabetes mellitus were at higher risk of developing tuberculosis in a large cohort recruited in an inner-city district with a high incidence for this outcome, and low socioeconomic conditions and high proportion of migrants. This risk was higher among Hindustan born and alcohol abusers.

## Introduction

Tuberculosis (TB) is a contagious airborne disease that has become the leading cause of mortality worldwide in recent years. The poorest countries and the most disadvantaged populations in developed countries are especially affected. According to the World Health Organization (WHO), in 2020, 10 million people developed this disease, causing 1,3 million deaths, 214,000 additional deaths in HIV-infected people. Two-thirds of TB cases worldwide are concentrated in 8 countries: India, China, Indonesia, the Philippines, Pakistan, Nigeria, Bangladesh and South Africa (1). At a global level, it is very difficult to calculate the number of cases with sub-diagnosis or with under notification. However, in the last WHO TB Annual Report, the data showed a substantial fall (18%) between 2019 and 2020, from 7.1 million TB cases to 5.8 million due to the impact of COVID. This set back, coupled with continued disruptions in 2021, mean that the United Nations high-level meeting target of treating 40 million people diagnosed with TB in the 5-year period 2018–2022 is off-track (1).

Regarding the situation of TB in Europe, high notification rates have been reported in big cities from low-incidence countries, including Birmingham, London, Brussels and Barcelona (2). In recent years, migratory movements to high-income countries from low-income countries with a high TB burden have changed the epidemiological pattern of this disease in recipient countries, mainly in disadvantaged urban areas of large cities (3). Additionally, these areas commonly have a high risk of overlapping health and social problems, contributing to TB incidence: homelessness, residents living below the poverty line and under overcrowded conditions, alcohol and drug abuse, and history of imprisonment (4, 5). An example of an inner district that meets these characteristics is *Ciutat Vella* in Barcelona, showing the highest incidence of TB in the city.

Diabetes mellitus (DM) is considered by the WHO as one of the most prevalent chronic diseases and one of the main risk factors for developing active TB (1). The global prevalence of DM is around 9.3% in adults between 20 and 79 years-old, affecting about 463 million people worldwide. According to recent estimates, the incidence of DM will continue growing (6), especially in areas with low and medium incomes and a high burden of TB, such as the Middle East, East Pacific, Southeast Asia and Africa, that represent 80% of the global burden of DM (6). In terms of notification, prevalence of DM was estimated to be around 13.8%, about half of that was unknown (7).

This increased risk of TB in DM patients may be, at least partly, explained by hyperglycemia-induced disruption of immunological mechanisms (8). Currently, there are new hypotheses about the role that hormonal and metabolic changes that take place in the context of diabetes would have in immunity against *M. tuberculosis* (9). Socioeconomic deprivation increases the risk of both conditions. Diabetic patients are much more frequently exposed to potentially contagious patients due to a higher rate of visits to healthcare facilities (10). In turn, TB can also become a risk factor for developing DM. Due to the inflammation induced by the infection itself, a transient hyperglycemia has been described in infected people at diagnosis (11), which can further be related to a greater risk of progression to DM (12).

Some studies have observed that the prevalence of latent TB infection (LTBI) is two times higher in diabetic patients (13). Also, diabetic subjects show an approximately three times higher incidence of active TB compared to non-diabetic subjects (14), more severe clinical presentation, with worse chest radiographs, more adverse effects to anti-TB drugs, a greater need for admission at diagnosis (15), and a higher risk of relapse after TB treatment (16, 17). Moreover, the risk of having sputum cultures remaining positive 2–3 months after starting the treatment would be approximately double. A two-fold higher risk of multidrug-resistant TB has also been observed among DM patients. Patients with DM and TB would have a 1.88 times higher risk of mortality (17, 18), especially those who are older or have more comorbidities or a lower albumin level (19).

The aim of our study was to determine the risk of TB in diabetic patients compared to a non-diabetic population in an inner-city district with a high TB incidence.

## Materials and Methods

### Study Design

We designed an observational matched retrospective cohort study (subjects with either type 1 or type 2 DM, and matched non-diabetic subjects). The recruitment period was between January 1st 2007 and December 31st 2016, with a follow-up period of two more years, until December 2018.

**Figure 1:**
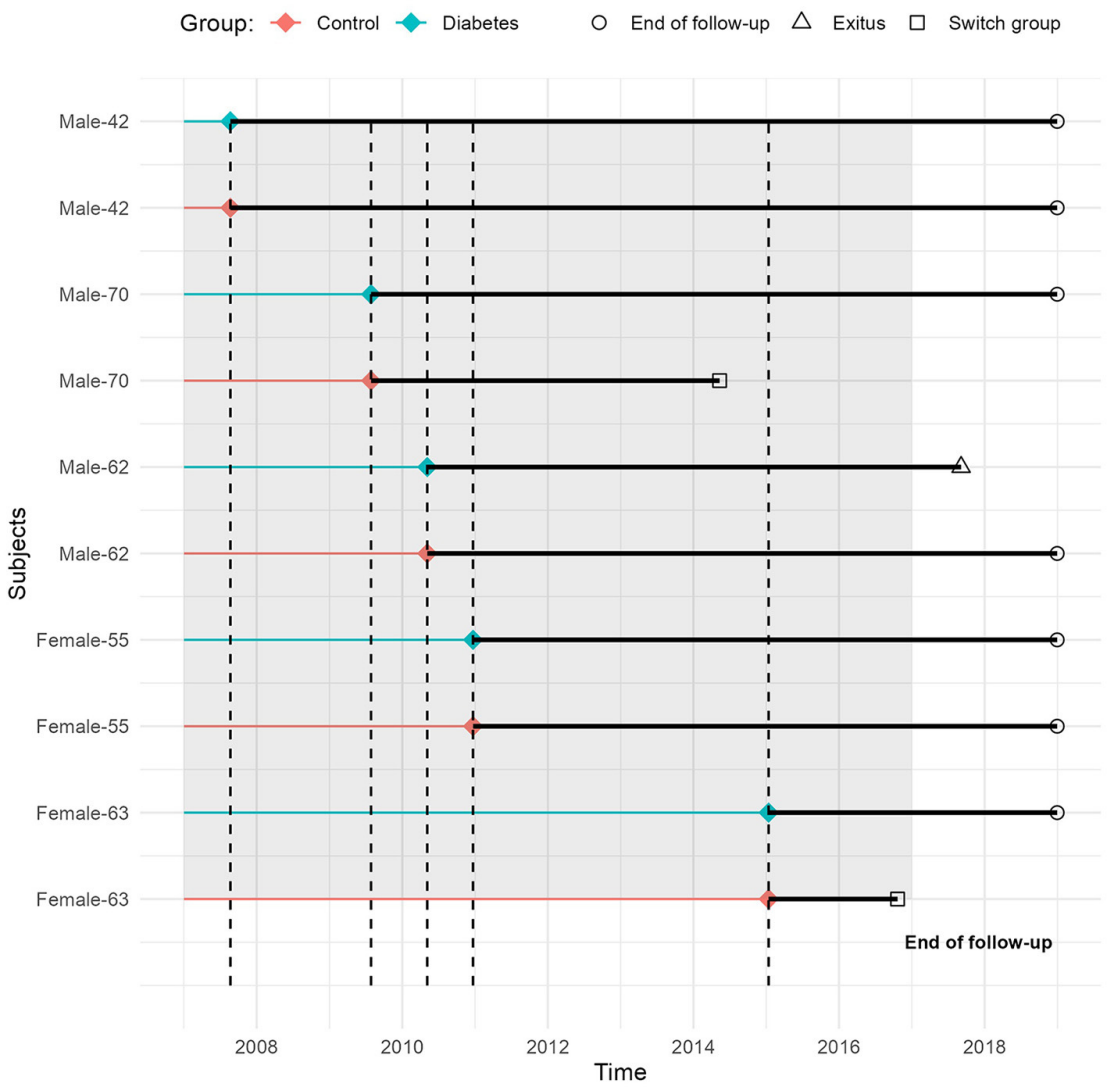
Sampling and follow-up scheme: an example of s pairs of individuals by age and sex.

The inclusion date was January 1st 2007 for prevalent diabetic participants, and the date of diagnosis for incident diabetic subjects during the recruitment period. For each DM case, a non-DM control matched by sex, year of birth, and time of inclusion was selected (ratio = 1:1), using a density sampling approach (Figure 1) see more information about the methodology in https://jrealgatius.github.io/TBC_ANALISIS/codi/shiny/DashBoard_TB.html.

### Study Population and Sample

The study was carried out in Ciutat Vella, a district in Barcelona (Northeastern Spain), which has been recently characterized by a high percentage of immigrants (50,1%) and a low socioeconomic level, with a population of 108,000 inhabitants (20).

**Figure 2:**
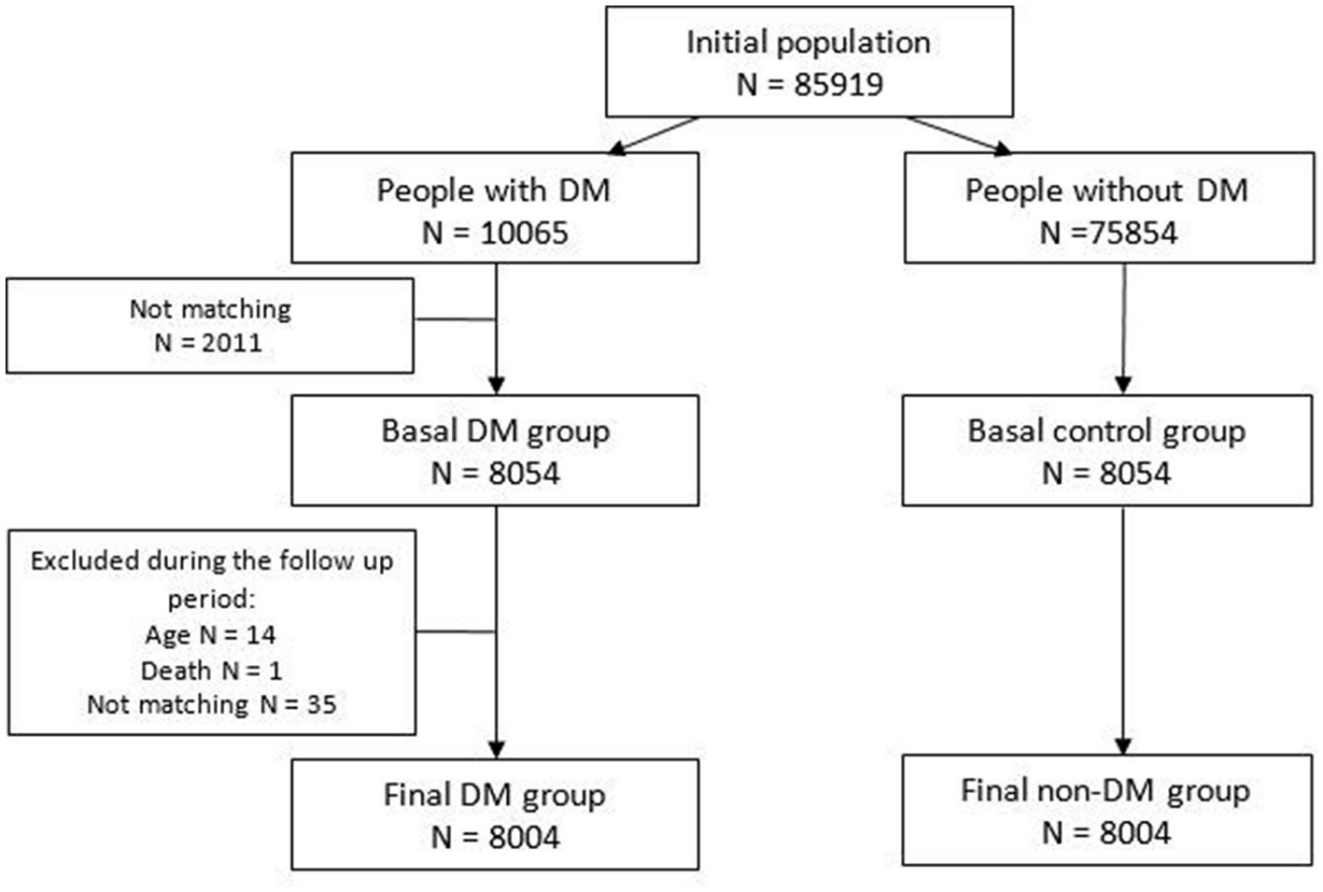
Flow chart of the cohorts within the study population.

All diabetic patients over the total attended population in Ciutat Vella were selected, and finally 8,004 of them were matched (see the flow-chart in Figure 2).

### Inclusion and Exclusion Criteria

In the DM cohort, patients aged over 18 years, fulfilling criteria for a DM diagnosis were included. The diabetic subjects were matched for age and sex with non-DM sample (controls).

People younger than 18 years old or without any appointment registered in their Primary Health Care Centre during the study period were excluded.

### Data Sources

All data was treated as confidential according to the ethical principles of the Helsinki Declaration of 1964, revised by the World Medical Organization in Edinburgh in 2000, and the Spanish Organic Law 3/2018 of Data Protection. Clinical characteristics and specific data on DM were extracted from the Primary Care medical record database from Barcelona. Specific data on TB were obtained from the Barcelona TB Prevention and Control program. Both sources were linked through an anonymized unique identifier. Because data was collected on a routine basis following the Spanish law about mandatory notification diseases, no informed consent was required. The data collection took place during 2019.

### Variables

The primary outcome was defined as the occurrence and date of a TB diagnosis during the follow-up period in DM and non-DM patients.

We obtained the following information on the subject at baseline: (1) demographic characteristics, smoking habit and excessive alcohol consumption (defined by the recorded International Classification of Disease 10 (ICE.10) F10.1), (2) clinical variables, (3) laboratory data, (4) history of comorbidities, (5) history of immunosuppressive or corticosteroids treatment, (6) history of sexually transmitted infections.

As Ciutat Vella is a district with a high percentage of migrants (50.1%), mostly from South-East Asia (Pakistan 13.6%, Bangladesh 8.1% and India 3.8%) (20), we grouped subjects coming from these countries in one category: Hindustani origin. Hindustan is an Asian peninsula formed by India, Pakistan, Bangladesh, Bhutan, Nepal, Sri Lanka and Maldivas.

We identified diabetic patients if they had an ICE 0.10 diagnostic code for DM in their medical record (E10–E14), or if they were taking any anti-diabetic drug other than metformin (sometimes used in other conditions). We also recorded glycated hemoglobin, DM duration, treatment (lifestyle management, oral medication and/or insulin), and microvascular complications: neuropathy, nephropathy or retinopathy.

A person was considered to have TB if they had a maintained anti-TB drug prescription according to the Barcelona TB Program; the program has an active epidemiological surveillance system with a good follow-up of patients. TB cases and their characteristics such as diagnostic procedure (smear observation, culture, tuberculin skin test), location (pulmonary or extrapulmonary), treatment and socioeconomic deprivation index (MEDEA) were collected. This index classifies the subject by living area: values have an average of 0 and a standard deviation of 1, and higher values indicate a more unfavorable socioeconomic situation (21).

### Statistical Methods

Baseline characteristics of both groups were evaluated to examine homogeneity in comorbidities and demographic and clinical characteristics, and were described by frequencies (n) and percentages (%), continuous and count variables were described using mean and standard deviation (SD), using compareGroups R Package (22). A complete data analysis was performed without imputation of missing data. If the diagnosis was not recorded, we assumed that the patient did not have the given condition. The main variables, DM and TB diagnoses, were based on clinical records. Continuous variables that were included in the models were categorized in tertiles, and the missing category was added.

We conducted a time-to-event analysis to estimate the incidence of TB by group, and to examine the association between DM condition and risk of TB. To examine time-related incidence curves and differences between groups, we performed competing risks regression for clustered data using crrSC R package version 1.1 (23). Risk functions and hazard ratios (HR) unadjusted and adjusted, with their 95% confidence interval (CI), were estimated. CIs and *p*-values were computed with robust standard errors to account for the matched sample. A sensitivity analysis of different fitted models was performed including different adjusted variables using a Cox proportional hazard model by clusters. The models were adjusted by origin and clinical characteristics with known clinical association with TB and DM. Statistical significance was established as a *p*-value < 0.05.

Finally, we performed a descriptive analysis of the cases of TB in DM and non-DM subjects. Data management and analysis was done with the R version 3.6.3 package (R Core Team 2020). The R data analysis scripts can be consulted at: https://github.com/jrealgatius/TBC_ANALISIS.

## Results

### General Characteristics

Initially we started from a potential population from Ciutat Vella (Barcelona) of 85,919: 10,065 diabetic subjects (prevalent and incidents during the follow up) and 75,854 potential non-diabetic subjects. Of these, 8,004 diabetics and 8,004 controls of the same age and sex were matched (Figure 2).

Regarding the sample demographic characteristics (Table 1), the average age was 57.7 years (SD = 14.2), and 61.2% were men and 38.8% women in each group. Twice as many patients in the DM cohort were of Hindustani origin (13.5 vs. 6.4% in the non-DM cohort). Diabetic patients had a higher frequency of TB-associated risk factors including alcohol abuse (5.0 vs. 3. 9% in the non-DM cohort), smoking (7.3 vs. 5.7%), chronic renal failure (3.2 vs. 1.1), corticosteroids treatment (1.1 vs. 0.6%), number of medical appointments (86.3 vs. 74.5) and a higher BMI (30.1 vs. 28.9).

**Table 1:** Baseline characteristics of the study variables between diabetic and non-diabetic cohorts.

**Variable**	**DM (*n* = 8,004)**	**Non-DM (*n* = 8,004)**	***p*** **-values**
Age: mean (SD)	57.7 (14.2)	57.7 (14.2)	0.921
**Gender:** ***n*** **(%)**			
Men	4,911 (61.1%)	4,914 (61.1%)	
Women	3,127 (38.9%)	3,127 (38.9%)	0.998
**Origin**			<0.001
Spain/high-income countries	5,949 (74.3%)	6,661 (83.2%)	
Hindustan	1,083 (13.5%)	508 (6.35%)	
Other	972 (12.1%)	835 (10.4%)	
Alcohol abuse	401 (5.01%)	311 (3.89%)	0.001
Smoking habit	588 (7.35%)	452 (5.65%)	<0.001
BMI	30.1 (5.30)	28.9 (5.02)	<0.001
SBP	137 (17.9)	134 (16.1)	<0.001
DBP	79.2 (11.2)	78.1 (10.8)	<0.001
Total cholesterol	207 (48.0)	212 (41.7)	<0.001
HDL cholesterol	48.1 (13.5)	54.4 (14.1)	<0.001
LDL cholesterol	124 (36.3)	133 (35.0)	<0.001
Triglycerides	106 (27.2)	99.2 (27.5)	<0.001
Hemoglobin	13.5 (1.25)	13.5 (1.20)	0.671
Leukocytes	11.5 (2.46)	12.0 (6.52)	0.501
Platelets	259 (71.5)	251 (69.7)	0.013
VSG	23.0 (19.4)	20.5 (16.7)	0.042
Chronic renal failure	254 (3.16%)	89 (1.11%)	<0.001
Kidney transplant	12 (0.15%)	5 (0.06%)	0.145
Peripheral arteriopathy	227 (2.84%)	94 (1.17%)	<0.001
Coronary heart disease	644 (8.05%)	280 (3.50%)	<0.001
Stroke	371 (4.64%)	177 (2.21%)	<0.001
Autoimmune disease^*^	80 (1.00%)	71 (0.89%)	0.513
Immunosuppressive treatment	36 (0.45%)	22 (0.27%)	0.087
Corticosteroids treatment	90 (1.12%)	49 (0.61%)	0.001
STI	51 (0.64%)	48 (0.60%)	0.840
AIDS	80 (1.00%)	158 (1.97%)	<0.001
Flu vaccination	3,039 (38%)	2,193 (27.4%)	<0.001
Number of medical visits	86.3 +/- 80.7	74.5 +/-77.6	<0.001
**Diabetic characteristics**			
Glycosylated hemoglobin: mean (SD)	7.41% (1.85)		
<7%: *n* (%)	1,818 (54.7%)		
>7%: *n* (%)	1,505 (45.3%)		
**Diabetes treatment**			
No treatment *n* (%)	4,824 (60.3%)		
Only oral medication *n* (%)	2,627 (32.8%)		
Insulin *n* (%)	553 (6.91%)		
**Diabetes complications**			
Diabetic nephropathy *n* (%)	95 (1.19%)		
Diabetic neuropathy *n* (%)	97 (1.19%)		
Diabetic retinopathy *n* (%)	297 (3.71%)		

* *Data are presented as number (percentage), mean ± SD (standard deviation); n, sample number; P value ≤ 0.05 were considered significant. AIDS, acquired immune deficiency syndrome; BMI, body mass index; DBP, diastolic blood pressure; DM, diabetes mellitus; HDL, high density lipoprotein; LDL, low density lipoprotein; SBP, systolic blood pressure; SD, standard deviation; STI, sexual transmitted disease; VSG, erythrocyte sedimentation rate. Autoimmune disease, Behçet, lupus, polyarthritis, rheumatoid arthritis, sarcoidosis, scleroderma, Sjögren, vasculitis*.

In the DM cohort, the mean DM duration was 3.2 years (SD 5.8), and 45.3% (n = 1,505) had a glycated hemoglobin value above 7% (Table 1).

### Risk of TB in DM Patients

**Figure 3:**
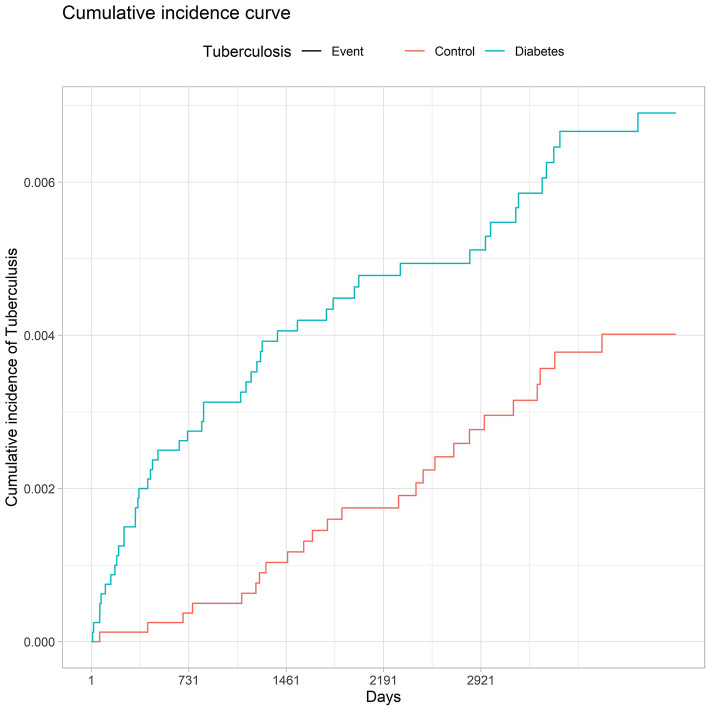
Incidence of tuberculosis in diabetic and non-diabetic patients.

At follow-up (Table 2), 73 new cases of TB had been reported (median follow-up = 8.7 years). The overall incidence rate was 56.2 cases per 100,000 inhabitants per year. Specific rates were 69.9 cases of TB per 100,000 person-years in the DM group, and 40.8 cases per 100,000 person-years in the non-DM group. The unadjusted risk ratio observed was 1.90 (CI: 1.18–3.07) (Figure 3).

**Table 2:** Incidence of tuberculosis in diabetic and non-diabetic subjects.

	**Number of subjects**	**Total person time (years)**	**Number of TB cases**	**Incidence density rate (95% CI)**	**Unadjusted risk ratio**	Adjusted hazard ratio^*^(95%CI)
Non-DM subjects	8,004	61,198.45	25	40.85 (26.44–60.33)	Ref	Ref
DM subjects	8,004	68,605.36	48	69.97 (51.59–92.76)	1.90 (1.18–3.07	1.66 (0.99–2.77)

* *Adjusted by: country of origin, chronic kidney disease, number of medical visits, BMI, alcohol abuse and smoking*.

The result of sensitivity analysis taking into account different adjusting variables and different approaches (competing risk models and Cox proportional hazard model by clusters) did not materially change the results in terms of the direction of the risk (Table 3).

**Table 3:** Estimated hazard ratios according to different approaches.

	**Variables in model**	**Competitive risk** **HR (95% CI)**	**Cox model** **HR (95% CI)**
Model 1	Group DM, origin	1.68 (1.03–2.75)	1.50 (0.92–2.45)
Model 2	Group, origin, number of visits	1.68 (1.03–2.74)	1.50 (0.92–2.45)
Model 3	Group, origin, number of visits, CKD; BMI	1.68 (1.01–2.79)	1.49 (0.90–2.46)
Model 4	Group DM, Origin, CKD, number of visits, BMI, alcohol, smoke	1.66 (0.99–2.77)	1.47 (0.89–2.44)

*BMI, body mass index; CKD, chronical kidney disease; HR, Hazard Ratio*.

**Figure 4:**
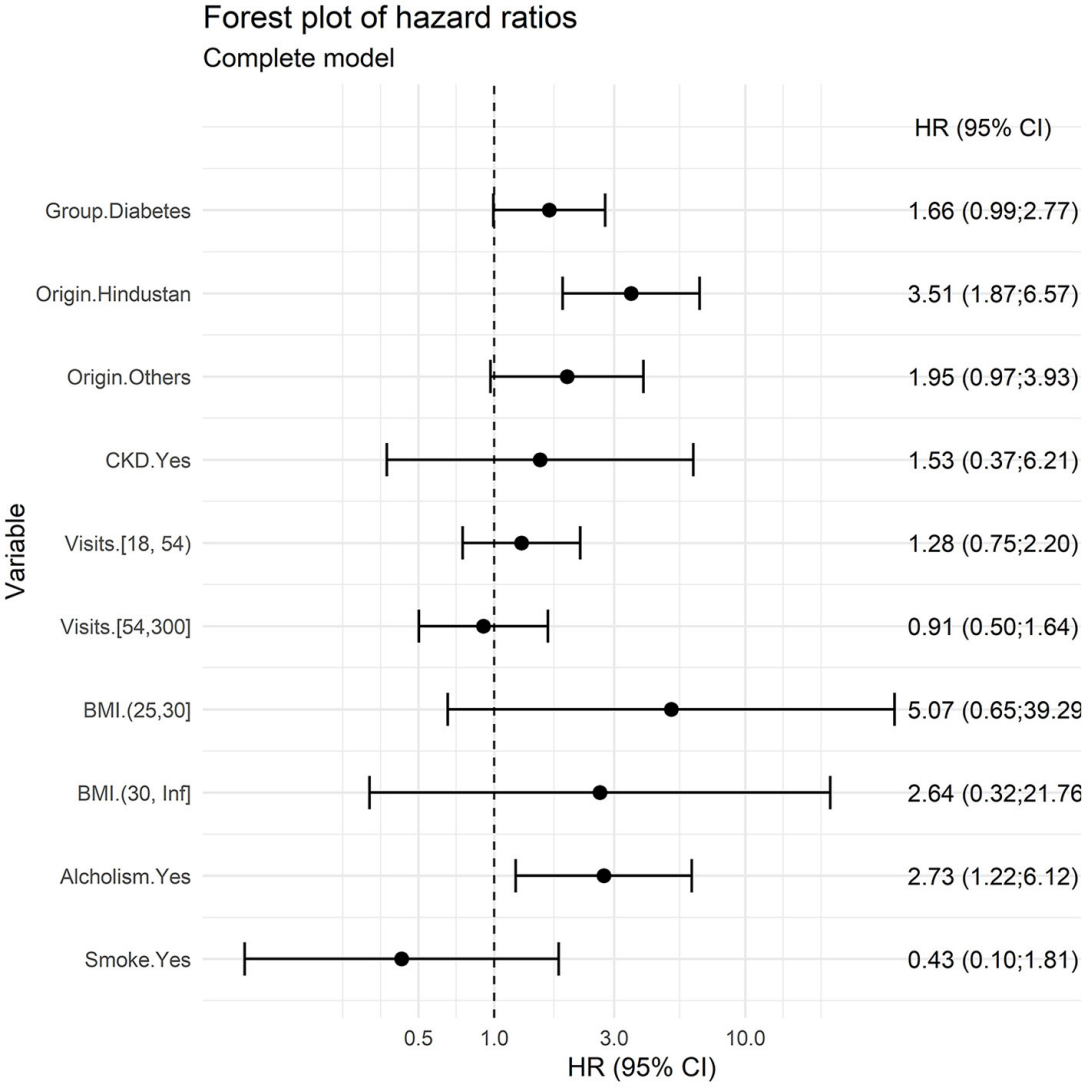
Forest plot of HR of each TB risk factor in the multivariate model.

The forest plot for different HRs of each analyzed variable related to TB (DM, country of origin, number of medical visits, chronic kidney disease, BMI, alcoholism and smoking) is shown in Figure 4.

### Differences Between DM and Non-DM Patients in TB Localization

Finally, we analyzed the differences between diabetic and non-diabetic patients who presented with a first episode of TB. Demographic and clinical data, including country of origin, smoking status, alcohol use, homelessness, unemployment, death, site of TB and tuberculin skin test, did not significantly differ between both groups. However, patients with TB from the two groups had a different socioeconomic deprivation index according to the Medea classification (Table 4). Regarding the location of the infection, we observed that in the DM group, 64.55% of the patients that developed TB had pulmonary involvement (with 6.25% of them with extrapulmonary involvement too), while in the non-DM group, 48% of the patients had pulmonary involvement, 12% with extrapulmonary implication.

**Table 4:** Demographic and clinical differences in TB patients according to their DM status.

	**DM (48)**	**Non-DM (25)**	***p*** **-value**
Origin			
Spanish-born	17 (35.4%)	12 (48%)	0.324
Foreign-born	31(64.6%)	13 (52%)	
Smoking	15 (31.2%)	9 (36%)	0.883
Alcohol abuse	8 (16.7%)	4 (16%)	1.000
Homeless	3 (6.25%)	3 (12%)	0.406
Unemployment	15 (32.6%)	4 (16.7%)	0.072
MEDEA (social risk)	1.55 (0.61)	1.88 (0.61)	0.048
Death	7 (14.6%)	1 (3.85%)	0.247
Localization			
Pulmonary	28 (58.3%)	9 (36%)	0.169
Extrapulmonary	17 (35.4%)	13 (52%)	
Both	3 (6.25%)	3 (12%)	
TST			
Unknown	1 (2.1%)	0 (0.0%)	0.844
Undone	25 (52.1%)	15 (60%)	
Negative	5 (10.4%)	2 (8%)	
Positive	17 (35.4%)	8 (32%)	

*TST, tuberculin skin test*.

*The percentages are referred to the total of each column*.

## Discussion

Our work showed a 1.90-fold higher risk of developing TB in DM patients. After adjusting for country of origin, chronic kidney disease, number of medical visits, BMI, alcohol abuse and smoking, the risk remained higher at 1.66 for DM patients. By analyzing other variables related to TB, our analysis revealed that subjects of a Hindustani origin and history of alcohol abuse were at higher risk for developing TB. As the use of alcohol is really frequent in our context (24), we considered using abuse of alcohol as a risk factor for TB, instead of use. When we classify the subjects under Hindustani origin, we refer to a geographical aspect. We consider that their country of origin, together with their migration process and living conditions in Barcelona, have a significant impact on their health status. Noteworthy, a higher proportion of DM subjects with TB had not been previously prescribed with any specific anti-DM treatment. We determined that the incidence rate of TB reached 56.24 cases/100,000 person-years, and most of the cases were diagnosed in foreign-born subjects (44 cases vs. 29 in Spanish-born patients). The number of subjects with TB was significantly elevated in foreign-born in both DM and non-DM cohorts. Interestingly, the incidence of DM was two-times higher in migrants coming from Hindustan compared to other origins. On the other hand, regarding the incidence of TB, DM patients had more pulmonary involvement compared to the non-DM group.

In the model 4, when adjusting by all covariables, the confidence interval remains in the limit of statistical significance, probably due to a lower statistical power or a risk of overadjustment. Hindustan origin and alcohol abuse are expected risk factors for developing TB because are also related to poor socioeconomic conditions in this context, and they are considered risk factors independently of having or not diabetes.

The inner-city district of Ciutat Vella is characterized by the lowest socioeconomic level in Barcelona and a high percentage of immigrants. Half of them are migrants from Asia (Pakistan 13.6%, and Bangladesh 8.1%), Oceania (Philippines 8,5%) and Central and South America (20). In 2017, the incidence of TB in this city district was estimated to be 43.8 cases per 100,000 inhabitants/year, while the global incidence in the city was 16.5 (25).

Our results showing a higher risk of developing TB in DM patients are in line with several other studies performed in different countries, with elevated estimated risk ratios being 1.77 times (CI: 1.41–2.24) for active TB in DM patients in China (26), 1.9 (CI: 1.7–2.1) in Denmark in 2015 (27), and 6 times (CI: 5–7.2) in southern Mexico in 2004 (28), to mention some. In addition, a systematic review of 13 observational studies from different countries published in 2008 reported 3-fold increased risk in subjects with DM (14). Co-existence of DM and TB has been described in studies done worldwide. In a report from Perú, DM was described as the most frequent comorbidity in patients with TB (29). In another work done in Ethiopia, DM had a high prevalence between patients with TB (30).

The higher incidence of TB in immigrant populations may be due to migrants coming from countries with a high incidence of TB. However, socioeconomic deprivation (i.e., living below the poverty line) and living conditions (i.e., overcrowded) may also increase exposure to TB. Indeed, in a study in people with TB from across Barcelona (2000–2013), autochthonous people had a higher risk of developing DM (15). In contrast, our work involved people from a deprived district where almost half of the inhabitants are migrants coming from high TB burden countries. According to another study, migration from outside the European Union (EU) does contribute to the TB burden, being one in five cases of TB notified in the EU originated in an extra-EU country between 2007 and 2013 (31).

Interestingly, an independent study conducted in 2008 in the same district, showed that Hindustani young people had up to 3 times more cases of type 2 DM than autochthon population, albeit having a lower average BMI (32). The notion that Asian Indians are more susceptible to develop type 2 DM is widely accepted (33), being probably due to a higher insulin resistance related to a central distribution of adiposity in these people. This is consistent with our results related to the higher incidence of DM found in Hindustani subjects.

Our data also showed that DM patients with TB had more pulmonary involvement. This result is consistent with previous reports (26, 34). In line with this, other presentations, such as osteoarticular, lymphatic and digestive involvement, have been highly associated with people of Indian subcontinent origin (35).

Because of the large number of subjects affected worldwide by both diseases, and that they exacerbate each other and increase the burden when they appear together, we can probably define this as a syndemic. Their interaction is a priority issue in global public health. TB is often related to poor living conditions and overcrowding, poverty and immigration. At the same time, these conditions are also related to poor nutritional habits, obesity and, in case of DM, poor glycemic control.

In contrast to DM, the contribution of obesity to TB is controversial. Although it is considered a risk factor for DM, it has been reported as a protective factor against TB development (36). An integrated approach, combining the management of the two diseases, could lead to a better health service for people in disadvantaged areas.

We believe that our work may be important to study the association of the two diseases and make specific strategic plans in areas of high TB concentration even in low-incidence countries.

As with chronic complications of the disease, glycemic control in DM patients probably influences both the risk (36) and the prognosis of TB (37). More studies are needed to analyse how the metabolic situation of DM can play a role in the different presentation and localization of TB, in treatment outcomes including mortality in patients with both conditions.

Some limitations can be found in our study. One of them is the retrospective and observational nature of the study design. All studies done with databases depend directly on the quality of the data record, which may be more worrisome in retrospective studies. In addition, retrospective studies may have more difficulty establishing a correct temporal relationship, and they have more selection and information biases, but we have tried to minimize these biases with a careful methodology and creating dynamic cohorts. Working with a population at risk of social exclusion may imply a risk of underreporting in key clinical factors. The 60.3% of DM without anti-DM treatment could be explained by a missing data in the databases or a lack of adherence to treatments. Moreover, some of the cases were incident DM; therefore, with the exception of cases with a highly elevated glycated hemoglobin, diet and exercise alone would have been recommended. However, the incidence rate shown was similar to the rates reported in independent studies (25). The measurement for glycated hemoglobin was sometimes lacking, as this is a retrospective study based in habitual medical practice. Apart from this, subjects' nationality, registered according to the Social Security data in the Primary Care medical record database, did not always correspond to the real origin of the subjects. Finally, there might have been missing data regarding evolution and treatment of TB, toxic habits or traveling.

Our work has also a number of strengths. Among these, the population database for a city area with an extremely high incidence of TB, and a long follow-up period of 10 years. Both sources of information are large and they provided important high quality information. Moreover, our results were consistent with the literature published elsewhere.

Further studies are required to investigate the interaction of both diseases, the contribution of optimization of glycemic control, and to consider whether screening for both diseases in specific areas could be cost effective.

## Conclusion

This large retrospective cohort study showed that in an inner-city district with high TB incidence and a high percentage of migrants, DM was associated with an increased risk of developing the disease. It is possible that the higher incidence was coupled with a higher TB transmission. Besides, TB in migrants can also be due to the reactivation of old infections, especially in people coming from Hindustan, where this disease is highly prevalent. Moreover, alcohol abuse was also identified as a risk factor for TB. In view of these results, we advocate for screenings to control diabetes-associated TB in the inner districts of high-income countries where the proportion of migrant people is high and characteristics such as low socioeconomic level further contribute to increase the risk.

Prevention of DM and TB are two of the WHO's main objectives. Changes in lifestyle, unhealthy diets, globalization, a greater access to diagnosis and a longer life expectancy could probably explain the increase of DM in countries where the burden of TB is greater.

We strongly recommend TB screening in DM patients in areas with a high TB incidence. According to the CDC's guidelines for the diagnosis of LTBI (38), Tuberculin skin test (TST) or TB blood test can be used indistinctly in diabetic patients. TB blood tests are recommended in people who are not likely to return for TST reading, have received the BCG vaccine, people who are likely to be infected with M. tuberculosis and are at a low to intermediate risk of progression to TB disease or people who have a low probability of being infected with TB. In case of using the TST in DM patients, a result of 5 mm or more should be considered positive.

## Data Availability Statement

Publicly available datasets were analyzed in this study. This data can be found here: https://jrealgatius.github.io/TBC_ANALISIS/codi/shiny/DashBoard_TB.html. The data analysis code is shown at https://github.com/jrealgatius/TBC_ANALISIS.

## Ethics Statement

The studies involving human participants were reviewed and approved by Ethics Committee of the Primary Healthcare University Research Institute IDIAP-Jordi Gol (P16/023). Written informed consent for participation was not required for this study in accordance with the national legislation and the institutional requirements.

## Author Contributions

VA-A, JC, JF-N, JR, ÀO, and AM-M participated in the study design. ÀO, JD, and JR worked on data collection. JR, VA-A, EN, RP, and JF-N performed all statistical work. VA-A, JF-N, and JC were major contributors in writing the manuscript. MM-C, DM, JJ, JM, JB, and AM-M reviewed and corrected the manuscript. BV contributed to prepare the manuscript according to the journal policies. All authors contributed to the article and approved the submitted version.

## Funding

This work was supported by two grants given by the Instituto de Salud Carlos III PI16/01751 (Spanish Ministry of Economy) and the Institut Universitari per a la Recerca a l'Atenció Primària de Salut Jordi Gol i Gurina (Catalan Health Institute) PREDOC_ECO-19/2. JJ was recipient of a Miguel Servet contract (CPII18/00004) (Instituto de Salud Carlos III).

## Conflict of Interest

The authors declare that the research was conducted in the absence of any commercial or financial relationships that could be construed as a potential conflict of interest.

## Publisher's Note

All claims expressed in this article are solely those of the authors and do not necessarily represent those of their affiliated organizations, or those of the publisher, the editors and the reviewers. Any product that may be evaluated in this article, or claim that may be made by its manufacturer, is not guaranteed or endorsed by the publisher.
